# Decoding and reasoning mental states in major depression and social anxiety disorder

**DOI:** 10.1186/s12888-020-02873-w

**Published:** 2020-09-24

**Authors:** Gheysar Maleki, Abbas Zabihzadeh, Mara J. Richman, Zsolt Demetrovics, Fatemeh Mohammadnejad

**Affiliations:** 1 Department of Clinical Psychology, Shahid Behashti University, District 1, Evin, Daneshjou Boulevard, Tehran, 1983969411 Iran; 2 Department of Psychiatry and Psychotherapy, Semmelweis University, Budapest, Hungary; 3 Endeavor Psychology, Boston, MA USA; 4 Department of Clinical Psychology and Addiction, Eötvös Loránd University, Budapest, Hungary; 5 Institute of Psychology, ELTE Eötvös Loránd University, Budapest, Hungary; 6 Orthopedic Research Center, Mazandaran University of Medical Science, Sari, Iran

**Keywords:** Major depressive disorder, Social anxiety disorder, Theory of mind, Decoding, Reasoning

## Abstract

**Background:**

Major depression (MDD) and social anxiety (SAD) disorders are debilitating psychiatric conditions characterized by disturbed interpersonal relationships. Despite these impairments in social relationships, research has been limited in simultaneously evaluating the dysfunction in MDD or SAD within two aspects of theory of mind (ToM): decoding mental states (i.e., Affective ToM) and reasoning mental states (i.e., cognitive ToM). Taking this into consideration, the current study assesses both decoding and reasoning mental states abilities in MDD, SAD, and healthy controls (HC).

**Methods:**

Subjects included 37 patients with MDD, 35 patients with SAD, and 35 HCs. ToM was measured with the Reading the Mind in the Eyes Test (RMET) and the Faux Pas Task, which assess decoding and reasoning mental states, respectively.

**Results:**

Results revealed that in decoding of mental states, both the SAD and MDD groups had lower scores than the HC group; there was no significant difference between the SAD and MDD groups in decoding mental states. Conversely, in reasoning mental states, the SAD and HC groups had higher scores than the MDD group; no differences were found between the SAD and HC groups.

**Conclusions:**

Clinicians and researchers should further consider parsing generalized impairment in ToM into two aspects: decoding and reasoning of mental states by using the aforementioned measurements. By further understanding the two aspects, we can create a potentially new clinical profile for mental health disorders, such as in this context with both decoding and reasoning mental state impairment in MDD and just a decoding impairment in SAD.

## Background

Theory of mind (ToM) has been recognized throughout the literature to be one of the most important characteristics of social cognition and has been defined as the ability to understand the mental states of others (i.e., wants, needs, beliefs, knowledge, emotions) [1–4] ToM is crucial in the everyday interactions of humans as it sustains social interactions through understanding the mental and emotional states of others [5–8] Many studies have found impairments in ToM in mental health disorders such as mood disorders [9–11] personality disorders [7], anxiety disorders [12], psychotic disorders [13, 14] and Alzheimer disease [15].

Singer [16] parsed ToM into two parts: affective ToM, or decoding mental states, (i.e., the attribution of emotional states to others) and cognitive ToM or reasoning mental states (i.e, as the understanding the intentions of others). Literature has extended Singer’s [16] theory by referring to affective and cognitive ToM as social-perceptual and social-cognitive aspects, respectively [17–19]. The social-perceptual aspect of ToM, also known as the “affective aspect,” refers to the ability to decode and discriminate the mental states of others based on available environmental information [18]. The social-cognitive aspect of ToM, or the “cognitive aspect,” refers to the ability to reason about mental states of others through interpretation or prediction of others’ behaviors [17, 18] The neurobiological substrates for the decoding phase of ToM have pointed to activation in the amygdala, the medial temporal structures, and the frontal lobe [18, 20] while the medial frontal area of brain has been identified as the most significant region corresponding to the reasoning phase of ToM [21, 22] In accordance with these differences in the decoding and reasoning aspects of ToM, recent studies have parsed out these two items, while evaluating dysfunction in both aspects of ToM in psychological disorders [4, 7, 22]

Research has found that anxiety and depressive disorders are not only one of the most highly prevalent psychiatric disorders, [2, 4] but also have impairment in successful and satisfactory interactions in individuals with depression and anxiety [23–26] Specifically, social functioning deficits are a common feature of Major Depressive Disorder (MDD) [10], which often contributes to the onset and continuation of depressive symptoms [24, 27] Considering the importance of ToM in social interactions, the evaluation of ToM ability in patients with MDD has been widely studied [9, 11, 25, 28] The results of these studies are consistent – those with MDD have dysfunction in ToM. In addition to MDD, findings have been shown in other psychiatric conditions comorbid with depression, this is the case. In a study by Zabihzadeh et al. [7], it was shown that patients with borderline personality disorder comorbid MDD have decreased ToM skills as compared to patients without comorbid MDD, pointing to a specialized profile. ToM deficits in depressed patients correlates strongly with impaired social abilities [8, 10] Since interpersonal conflict is an integral element in symptom onset of depression [27], ToM impairment is a predictor in depression reoccurrence. Many studies have evaluated ToM in patients with MDD, but only two studies (Wang et al. [11] & Wolkenstein et al. [9]) have simultaneously investigated decoding and reasoning aspects of ToM. According to Wang et al. [11], MDD patients had decreased performance in both decoding and reasoning dimensions of ToM as compared to healthy controls. On the other hand, the results of Wolkenstein et al. [9] indicate that MDD patients have decreased performance only in reasoning mental states in comparison to healthy controls, while the performance of these patients in decoding mental states was not impaired. Both studies used the Reading in the Mind of the Eyes Test (RMET) [28] to measure decoding mental states. Considering the inconsistencies, further studies measuring the simultaneous measurement of decoding and reasoning aspects of ToM in MDD patients is of importance.

Despite the large amount of studies on ToM in patients with depression, little research has assessed the ability of ToM in anxiety disorders, such as social anxiety disorder (SAD). SAD is a psychiatric disorder characterized by persistent, excessive fear, and avoidance of social and performance related situation [29] sand is a chronic and debilitating psychiatric condition, leading to social and interpersonal impairments [25]. Previous studies have proposed that high levels of social anxiety may be attributed to social cognitive deficits, which are manifested toward inaccurate and distorted appraisals of the beliefs and intentions interpersonally [30, 31] Despite social and interpersonal impairments, only three studies have assessed ToM ability in SAD. According to Samson et al. [32], the high scores on the social anxiety scale are associated with decreased ToM ability, however, individuals with social anxiety in the non-clinical range. Furthermore, in this study, ToM ability was evaluated only with cartoons that involved the interpretation of others’ mental states. This task is most common in the measurement of reasoning ToM but not for the decoding aspect. Moreover, Hezel & McNally [33] found that SAD patients compared to the healthy control group had lower performance in ToM tasks (within the RMET).

Washburn et al.’s [25] study is the only study that considered ToM abilities in the clinical case of SAD patients. The results of this study which compared ToM in SAD and MDD patients with and without comorbid depression, demonstrated that the group of non-comorbid SAD patients had significantly lower performance in comparison to the healthy control and non-comorbid MDD groups. Furthermore, both the comorbid and non-comorbid SAD groups made significantly more ‘excessive’ ToM reasoning errors than the non-comorbid MDD group, suggesting a pattern of over-mentalizing. Though evaluation of ToM ability in both the RMET [34] and in the movie for the assessment of cognition (MASC) [35] was used, the main goal of the study was not to differentiate the performance of patients in decoding and reasoning aspects of ToM. For this reason, the results of this study were not discussed based on the relationship between the performance of patients in ToM tasks with the decoding and reasoning aspects.

The reason for our study was to simultaneously assess decoding and reasoning aspects of ToM in MDD, SAD, and healthy controls (HC). We hypothesized that impairments of ToM in MDD is more severe than in SAD. Furthermore, we expect that the HC group (in both decoding and reasoning mental states) is better than the MDD and SAD groups.

## Methods

### Participants

The participants included three groups: patients with MDD (*n* = 37, 54.05% female, mean age: 28.17, SD: 2.27), patients with SAD (*n* = 35, 54.28% female, mean age: 27.49, SD: 2.06) and HC group (n = 35, 48.57% females, mean age: 28.38, SD: 3.41). Patients with MDD and SAD were recruited from four psychological services clinics in Sari, Iran. Patients were diagnosed with MDD or SAD according to the Structured Clinical Interview for DSM-IV for Axis I Disorders (SCID-I) [36].

Exclusion criteria for the two patient groups were the following: a) any current or past diagnosis of a psychotic disorder, b) autism spectrum or any developmental disorders, c) bipolar disorder and/or d) any neurological diseases such as epilepsy, Parkinson’s disease, or severe head injury. Individuals were also excluded if they had any substance abuse issues history during the preceding 6 months. Patients in the SAD group were excluded if they had any history of major depression, and in the MDD group, patients were excluded if they had a history of SAD.

The healthy control (HC) group was recruited from the Islamic Azad University in Sari, Iran. None of the participants in the HC group had a history of any DSM-IV Axis I or Axis II disorders as verified with SCID-I, a brain injury, neurological diseases, and/or evidence of current or past substance abuse.

All participants were included if they had the following criteria: a) at least 20 years old, b) able to understand the experimental procedure, and c) had normal visual and auditory senses.

### Clinical assessment

Diagnoses were completed using the Persian version of Structured Clinical Interview for DSM-IV Axis I Disorders (SCID-I) [36]. In addition, the SCID-II was for Axis II disorders. All subjects filled out the Persian version of Beck Depression Inventory-II (BDI-II) [37] and the Persian version of the Beck Anxiety Inventory (BAI) [38] which looked at the severity of depression and anxiety symptoms, respectively. The BDI is a 21-item self-report measure developed to assess the attitudes and clinical symptoms in both depressed and non-depressed psychiatric patients [39], whereas the BAI is a 21-item self-report measure, which evaluates the severity of anxiety symptoms [40]. The Persian versions of the BDI-II and the BAI have been reported to have good psychometric properties [37, 38] We also used the Wechsler Adult Intelligence Scale-Revised Version (WAIS-R) [41] to assess overall intellectual functioning.

### TOM tasks

#### Reading in the mind of the eyes task

In order to asses mental state decoding, we gave participants the Reading in the Mind of the Eyes test (RMET), [34] which as translated into Persian [42]. The RMET included 36 black-and-white photographs of eyes (15 cm × 6 cm). Of that, items were are emotionally valenced (i.e., neutral, negative, and positive), and the participants are asked to select which emotion best matched the picture in valence. Those involved were required to express their opinions on the gender of each picture (i.e., gender recognition). There was no time limit for answering questions, and the total score was calculated based on the total participant’s correct response to each picture; the highest score a participant could acquire was 36. Following the studies of Harkness et al. [43] and Richman & Unoka [28], three subscales were also calculated based upon the value of each mental state (i.e., 8 positive, 12 negative, and 16 neutral). Previous studies have indicated attentional bias to negative stimuli in depressed individuals [44, 45]; therefore, in this study, accurate differentiation of the three groups in discrimination of positive and negative mental states was used

#### Faux pas task

The faux pas task was used to assess mental states reasoning. The test had 20 short stories; half of them comprised of a faux pas while the other half included control stories. Baron-Cohen et al. [46] states that a Faux Pas occurs when a speaker mentions something without thinking about if the listener might not want to hear or know with negative consequences (See Appendix 1). No time limits are considered. When every story ended, there were two faux pas questions organized with two control ones. The faux pas questions looked at the main character’s intentions and were put together to assess whether or not they could distinguish if a faux pas had taken place.

Control questions were designed to check the reader’s understanding of the story. Subjects who replied “yes” to the first question (e.g., In the story you just read, has there been a faux pas and/or an embarrassing mistake in a social situation?) were also told to answer the subsequent faux pas question; meanwhile, in the stories involving a faux pas, one score was for each correct response. When the participant’s answer to the first question was “no”, they were not necessitated to answer a follow up question; however, all participants were told to answer two control questions, even if their answers to the first question had been negative. In the end, 20 was the maximum score a participant could get on the Faux Pas Questions, and 40 on the control.

### Procedure

Following a brief description of the study, participants provided written informed consent. Then, all participants underwent a demographic questionnaire and the SCID-I. Next, they were dividied between tasks: half of the participants completed RMET before Faux Pas, and the other half first completed Faux Pas then RMET.. After the ToM tasks, participants completed the BAI and BDI-II. All tasks were administered in a single session and lasted approximately 120 min. The ethics committee of the Faculty of Psychology and Education of Shahid Beheshti University approved the procedure.

### Statistical analysis

All statistical analyses were performed using SPSS 23. We computed frequency and mean scores for the participants’ demographic and clinical data. Before using parametric tests, we used Kolmogorov–Smirnov and Levene’s tests to assess normality and homogeneity of variance for demographic, clinical, and ToM tasks scores. A Pearson’s Chi-squared test was performed to analyze the sex ratio of the groups and a parametric one-way analysis of variance (ANOVA) was employed for index age, educational level, IQ, and clinical measures (BDI-II and BAI). Two one-way multivariate analysis of variance (MANOVA) with post-hoc Tukey comparisons were used to compare groups in decoding ToM (RMET and its subscales) and reasoning ToM (Faux pas). Disorder type (SAD, MDD or HC) was included as an independent variable; RMET and Faux-pas scores as dependent variables. For all analyses, the level of statistical significance was set at *P <* 0.05.

## Results

### Demographic and clinical data

Demographics of the groups can be seen in Table 1. There were no significant differences between the participants in the three groups in terms of mean age, educational level, and IQ; however, when comparing the clinical data and BDI-II scores, the difference between groups was significant (*F*(2, 104) = 40.36, *p* < 0.001). Post hoc comparisons showed that MDD and SAD groups had higher scores than the HC in BDI-II scores. Moreover, the SAD group had lower scores than the MDD group in BDI-II. In BAI, three groups had also significant difference (*F*(2, 104) = 38.12, *p* < 0.001). The two patient groups had higher scores than the HC in BAI. Also, the MDD group had lower scores than the SAD group in BAI (see Table 1).

**Table 1:** Comparisons of demographic data and clinical data among groups

	HC (*n* = 35)	MDD (*n* = 37)	SAD (*n* = 35)	Statistics
Sex ratio (M: F)	20:17	17:20	16:19	χ2 = 0.58, *P* = 0.37, n.s.
Index age (years)	28.38 ± 3. 41	28.17 ± 2. 27	27.49 ± 2.06	F = 1.39, *P* = 0.11, n.s.
Education levels (years)	16.21 ± 2.09	14.78 ± 2.35	14.36 ± 1.70	F = 1.08, *P* = 0.61, n.s.
IQ	110.48 ± 5.80	107.29 ± 7.61	108.52 ± 5.20	F = 1.46, *P* = 0.41, n.s.
BDI-II	8.11 ± 3.28	38.12 ± 3.84	20.36 ± 5.11	F = 40.36, *P* = 0.001 MDD > HC, MDD > SAD, SAD>HC
BAI	7.62 ± 3.39	19.71 ± 4.27	41.59 ± 6.13	F = 38.12, *P* = 0.001 MDD > HC, MDD < SAD, SAD>HC

### Comparisons of decoding ability among three groups

Multivariate analysis of variance (MANOVA) revealed a significant difference between the three groups in the total score of ToM (*F*(2, 104) = 11.27, *p* < 0.001). Tukey post-hoc comparisons indicated that the HC group performed better than the SAD and MDD groups; moreover, there was no significant difference between the SAD and MDD groups in the total score of ToM. In ToM subscales, the difference between the three groups was significant. In positive valence (*F*(2, 104) = 8.19, *p* < 0.001) and neutral valence (*F*(2, 104) = 13.71, *p* < 0.001), the HC group had higher scores than the SAD and MDD groups. For negative valence, the HC group had lower scores compared to the SAD and MDD groups (*F*(2, 104) = 7.48, *p* < 0.001). Moreover, there was no significant difference between the two patient groups in ToM subscales. In gender recognition, no significant differences were found between the three groups (*F*(2, 104) = 0.24, *p* < 0.84) (Table 2).

**Table 2:** Comparisons of decoding and reasoning mental states among groups

Measures	MDD (*n* = 37)		SAD (*n* = 35)		HC (*n* = 35)		F	*P*	Partial η2	Post hoc
	M	SD	M	SD	M	SD				
Total ToM	23.81	3.29	24.94	3.11	27.60	3.74	11.72	0.001	0.18	HC > SAD & MDD
Positive ToM	5.32	1.49	5.71	1.21	7.37	2.09	8.19	0.001	0.16	HC > SAD & MDD
Negative ToM	9.15	2.39	8.92	1.83	6.10	1.69	7.48	0.001	0.15	HC < SAD & MDD
Neutral ToM	9.34	2.54	10.31	2.47	14.13	3.28	13.71	0.001	0.22	HC > SAD & MDD
Gender Recognition	32.37	2.39	31.54	2.31	32.45	2.63	0.24	0.84	0.002	n.s
Faux pas	13.45	2.51	16.02	2.09	16.71	1.74	23.16	0.001	0.30	MDD < SAD & HC
Control	37.16	2.03	36.74	1.96	36.77	2.08	0.48	0.61	0.009	n.s

### Comparisons of reasoning ability among three groups

**Fig. 1:**
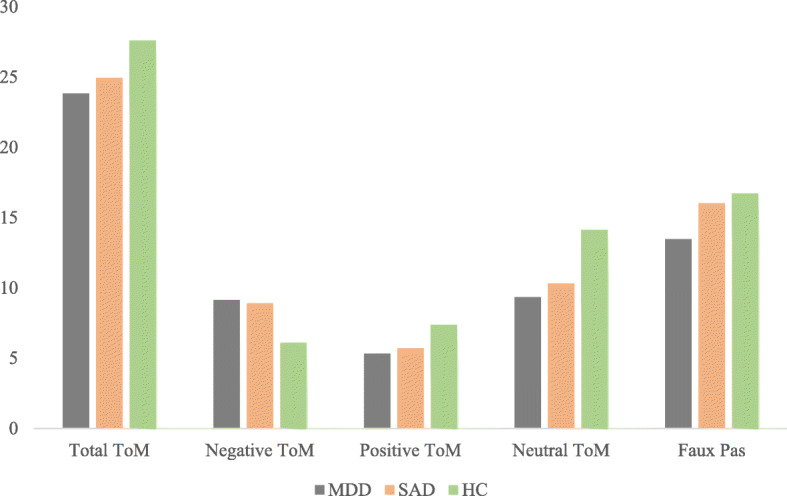
Mean of accurate responses of RMET and Faux Pas test in three groups. HC: Healthy Controls; MDD: Major Depression Disorder; SAD: Social Anxiety Disorder; BDI-II: Beck; Depression Inventory; BAI: Beck Depression Inventory; n.s.: not significant difference

Results of the MANOVA indicated a significant difference between groups in the Faux pas test. The Tukey Post hoc comparison showed that the SAD group had higher scores than the MDD group (*F*(2, 104) = 23.16, *p* < 0.001). There were no significant differences between the three groups in the control questions (*F*(2, 104) = 0.48, *p* < 0.61) (Table 2). Fig. 1 illustrates the performance of the three groups on the RMET and Faux Pas tests.

## Discussion

The current study compared mental state decoding and reasoning in SAD, MDD, and HCs. We included clinical cases of SAD, which is not often presented in other studies. The results regarding the decoding aspect, measured by the RMET, demonstrated that both groups (SAD and MDD) had decreased functioning than the HC group, while there weren’t significant differences between these two patient groups otherwise. The decreased functioning of MDD patients in the RMET is consistent with previous studies [8, 10, 26, 28]

According to results of the current study, the MDD patients not only suffer from deficits in mental states decoding ability, however, in comparison to SAD patients and healthy groups had lower performances in the reasoning aspect of TOM; however, in the reasoning ability, SAD and the healthy groups had no significant differences. In fact, the decreased functioning of the MDD patients in both decoding and reasoning mental states indicated the general impaired of ToM in these patients. The recent findings are consistent with Wang et al. [11] as our results demonstrated that depressed patients have more impairments in the decoding and reasoning dimensions of ToM. On the other hand, our findings are inconsistent with the results of Wolkenstein et al [9] Based on Wolkenstein et al. [9], MDD patients, in comparison to HCs, had normal functioning in RMET, however, they demonstrated lower functioning in the MASC test; the findings of normal functioning in the RMET is inconsistent with other related studies [8, 10, 26, 28] It seems that this discrepancy originated from the small sample size in Wolkenstein et al. [9] which only 24 MDD patients were compared with 20 healthy controls. However, other related studies have sample sizes at around 30 participants. Furthermore, another reason behind this inconsistency can be related to the difference in the severity of depression in the samples of previous studies. Lee et al. [47] concluded that individual differences in the severity of depression differentially predict ToM among depressed individuals. Thus, those who are severely depressed will be more impaired on ToM tasks than those with a mild to moderate level of depression. As mentioned above, Wolkenstein et al. [9] found that MDD patients are capable of decoding and distinguishing correct mental state of others, however, they are impaired in mental state reasoning. Results of the current study are inconsistent with Wolkenstein et al. [9] while they are consistent with Lee et al. [47].Lee et al. found that MDD patients widely suffer from ToM impairment in the case of decoding and reasoning aspects. In previous studies, it’s been suggested that when depression is comorbid to another disorder, dysfunction in ToM is increased. These results demonstrate that MDD patients have difficulty in social interactions, which is associated with chronicity and functional decline [48, 49] These difficulties are representations of generalized impairments in ToM in MDD patients, which is highly associated with the dysfunction of social interaction skills. One of the major reasons for this association are the same brain structures engaged in ToM and depression. The literature suggests the crucial role of ToM regions in the pathophysiology of depression. The available studies demonstrate that the prefrontal, orbitofrontal, ventromedial prefrontal cortex relate to the neural underpinnings of ToM [49, 50] Furthermore, the neuroimaging studies indicated that the prefrontal cortex plays a critical role in the pathophysiology of mood disorders [49, 51]. Some studies have shown that MDD patients have a smaller volume of orbitofrontal cortex in comparison with normal individuals [52, 53] These findings elucidate the role of this brain region in pathophysiology of MDD. On the other hand, others have regarded the orbitofrontal cortex [54] as having a role in the recognition of mental states through eye region photographs.

Unlike the generalized impairments of ToM in MDD patients, which involves both decoding and reasoning aspects, SAD patients only had difficulty in decoding mental states or the affective aspect. Based on our knowledge, the current study is the first piece of research that has simultaneously differentiated function of SAD patients in both decoding and reasoning aspects of ToM. The low function of SAD patients in RMET is consistent with the study of Hezel & McNally [33] as well as Washburn et al [25] An interesting point of this study is that unlike MDD patients, SAD patients did not have any significant differences as compared to HCs in the faux pas test (i.e., reasoning aspect) of ToM. Also, it should be noted that SAD is generally accompanied by MDD, and the clinical characteristics of MDD are more severe in SAD [25]. Therefore, it can be expected that the severity of impairments of ToM in MDD is higher than in SAD patients. Longitudinal studies have indicated that patients who suffer from SAD who later are diagnosed with MDD are characterized by higher levels of interpersonal over-sensitivity and social impairment than those who do not [55, 56] According to the results of this study, impairment of ToM in SAD patients is solely representing its decoding ability of mental states, and these patients are not suffering from the dysfunction in reasoning ability. Therefore, the impairment of interpersonal interaction of SAD patients is a result of their impairment in the decoding aspect of ToM. According to attention control theory [57], anxiety causes impairments in the attention system and can lead to a decrease in the attention control and then excessive attention to threatening stimulants. Difficulty in inhibition and shifting of attention would probably lead to difficulty in recognition, decoding, and understanding the cognitive realization of others in anxious individuals. It is interesting that the results of the current study in the subscales of RMET in both SAD and MDD patients are aligned with cognitive perspectives in the attentional bias of patients to negative stimuli. In this study, MDD and SAD patients in comparison to the healthy group had lower scores in total ToM but higher scores in the negative subscale. The results are consistent with the findings of Wolkenstein et al. [9] in which the MDD patients represented higher function than HC in recognition of negative mental states. Cognitive models of depression emphasize that depressed individuals tend to negatively interpret the vague stimuli; these biases are crucial in the initiation and persistence of the disorder [58]. Depressed individuals tend to interpret social situations negatively, and they have a better memory for negative stimuli. This deficit is strongly consistent with the dysfunction of social interaction skills in SAD patients [44]. Furthermore, the inability to divert attention from threatening stimuli and to shift it to other stimuli [57] can also be regarded as the probable reason for the weakness of the SAD patients in decoding positive and neutral mental states. On the other hand, that inability causes high functioning in recognition of negative mental states. Related previous studies [25, 33] did not demonstrate a distinction between the recognition of negative, positive, and neutral mental states in SAD patients.

In this study there are some limitations. The first limitation of this study is that we did not measure the number of episodes and relapse rate in MDD patients. There is some evidence regarding the relationship between ToM and the higher rates of relapse in depressed individuals [59], so investigation of this relationship is important. Second, SAD and MDD patients were the ones seeking treatment. It would be better if a wider range of SAD and MDD patients were examined so that a more reliable conclusion can be reached regarding their ToM ability. Third, in this study the possible effects regarding the use of medication on performance of SAD and MDD patients was not analyzed. In addition, we used a categorical approach for the diagnosis of SAD and MDD, whereas a dimensional model allows for varying degrees of severity that may increase the validity of a diagnosis [29]. Finally, we were limited by our small sample size.

## Conclusion

Overall, the findings of this study confirmed that there is a generalized impairment in ToM in MDD patients, and that the SAD group only had a deficit in the decoding aspect of ToM. Together ToM impairments can contribute to a dysfunction in social communication skills especially in MDD patients and have some important implications for clinicians regarding the implementation and planning of psychotherapeutic interventions.

## Data Availability

The datasets analyzed during the current study are available from the corresponding author on reasonable request.

## Appendix 1

Nahid had just moved into a new apartment. Nahid went shopping and bought some new curtains for her bedroom. When she had just finished decorating the apartment, her best friend, Zahra, came over. Nahid gave her a tour of the apartment and asked, “How do you like my bedroom?” “Those curtains are horrible,” Zahra said. “I hope you’re going to get some new ones!”

Did anyone say something they shouldn’t have said or something awkward?

**If yes**, ask:

Who said something they shouldn’t have said or something awkward?

**Control question**:

In the story, what had Nahid just bought?

How long had Nahid lived in this apartment?

## Abbreviations

BAI: Beck Anxiety Inventory
BDI-II: Beck Depression Inventory-II
HC: Healthy controls
MANOVA: Multivariate analyze of variance
MASC: Movie for the assessment of cognition
MDD: Major depression
RMET: Reading the Mind in the Eyes Test
SAD: Social anxiety disorders
SCID-I: Structure Clinical Interview for DSM-IV Axis I Disorders
ToM: Theory of mind
WAIS-R: Wechsler Adult Intelligence Scale-Revised Version
